# Assessment of burden and needs of family caregivers for the elderly, a scoping review

**DOI:** 10.3389/fragi.2025.1578911

**Published:** 2025-06-13

**Authors:** Elodie Le Toullec, Anne Le Gagne, Emilie Leblong, Alain Somat, Patrice Piette

**Affiliations:** ^1^ Fondation St Hélier, Departement Clinical Research, Lab St Helier, Rennes, France; ^2^ Department Human Sciences, LP3C, Rennes 2 University, Rennes, France

**Keywords:** family caregivers, assessment, elderly, burden, needs, wellbeing, psychometric properties, chronic stress

## Abstract

**Background:**

Family caregivers play a central role in supporting older adults. Their role is complex and challenging. Their assessment is essential for planning support systems. This review aims to catalog the different scales, identify the spectrum of dimensions they cover, and evaluate their psychometric quality, with the aim of reflecting on potential clinical recommendations.

**Method:**

A systematic review of MEDLINE, PsycINFO, google scholar and CINAHL databases identified tools for measuring family caregiver engagement. Two reviewers selected relevant studies using predefined criteria. Keywords included “family caregivers,” “engagement,” “measurement,” and “psychometrics.” Extracted data was analyzed for reliability, validity, and other psychometric properties.

**Results:**

We identified 140 articles, 38 of which were analyzed, utilizing 29 different scales to assess caregiver support across various dimensions such as burden, stress, or needs. Clinimetric assessment using an abbreviated COSMIN checklist revealed significant variability in the reliability and validity of these scales, with only 2 meeting clinical research standards. Notable deficiencies were found in internal consistency, reproducibility, and construct validity. None of the scales cover the entire range of complex dimensions associated with family caregivers.

**Conclusion:**

The study underscores the need for improved measurement tools tailored to the complexities of caregiver engagement. Future work should focus on developing more refined scales that better capture the diverse needs of family caregivers to enhance support.

## Introduction

The evolution of the global population shows a trend of a steady increase in the number of elderly people, thanks to advances in medicine that extend life expectancy. INSEE estimates that one-third of the French population will be over 60 years old by 2060 (Gand, Hénaut and Sardas, 2014). People over 85 years old, currently considered the “fourth age,” will number nearly 5 million by then (Gand, Hénaut and Sardas, 2014).

Among the consequences of socio-demographic transformation is the increased occurrence of chronic degenerative physical or cognitive diseases, such as dementia, recognized as the leading cause of disability among the elderly (Maresova et al., 2019) and osteoarthritis (Di Nicola, 2020). These diseases have a serious negative impact on an individual’s quality of life. They cause pain, reduced mobility, and functional, psychological, and social impairments. They can also lead to depression (Naselli et al., 2023).

As the severity of age-related deficits or illnesses increases, elderly individuals frequently require the assistance of a primary caregiver (Wong, Gilmour and Ramage-Morin, 2014). In France, 9.3 million people provide daily support to a loved one who has lost autonomy or is disabled (Drees, 2023). Eighty percent of the daily help provided to elderly people comes from their relatives, primarily spouses or children (Maresova et al., 2019). Among them, 23.5% are aged between 60 and 65 years old (Drees, 2023). In 2021, there were approximately 8.8 million adult and 500,000 minor caregivers in France (Agir pour les aidants, 2023).

The support from caregivers represents the fundamental cornerstone of care (Mace and Rabins, 2017). They play a strategic role in the lives of the people they help and, more broadly, in maintaining the elderly’s independence at home (Gayle et al., 2016). Having the support of a caregiver allows for delaying the need for formal home care services or entry into nursing homes (Mittelman et al., 2006).

Caregivers, also referred to as family caregivers, informal caregivers, or natural caregivers, are individuals who provide regular and frequent non-professional assistance to support and perform daily activities for individuals experiencing a loss of autonomy due to age, illness, or disability (Perissinotto et al., 2012). This assistance can be provided independently or as a complement to the work of a professional home care worker, such as a caregiver, home helper, nurse, or social worker. This help can be provided regularly, whether continuously or at more spaced intervals.

### Role and involvement of caregivers

Caregivers play a crucial role in supporting “vulnerable” individuals, often at the expense of their own personal, professional lives, health, and wellbeing (Agir pour les aidants, 2023; Schulz and Eden, 2016). This role demands a combination of skills, commitment, and physical endurance that can be difficult to maintain over time. The quality of life for both the care recipient and caregiver can be affected—care recipients by the care they receive, and caregivers by the intensity of their tasks.

From the perspective of care recipients, individuals with dementia who live at home with a caregiver tend to have a higher quality of life than those in 24-h care facilities (Hoe et al., 2007). Additionally, the quality of the relationship between the caregiver and care recipient has been shown to predict the care recipient’s quality of life and psychological wellbeing (Burgener and Twigg, 2002). French cohort studies have also highlighted the effects of caregiving on the health and morale of caregivers (Thomas et al., 2011).

From the caregiver’s perspective, family members often play a key role in supporting loved ones with dementia, acting as protectors and care coordinators (Bunn et al., 2017). Caregivers identify problems, seek help, and participate in medical decisions. They also manage appointments, organize transportation, and relay information between healthcare professionals, ensuring continuity of care. The presence of a family caregiver is essential for accessing care, especially for disabled individuals living alone. Most often, the elderly person and the caregiver face confusing and disconnected care systems, involving a range of entities, including healthcare providers, public and private community organizations, employers, and multiple potential payers. Caregivers must navigate these multiple, evolving, and increasingly complex systems, often without assistance (Schulz and Eden, 2016).

Caregivers, however, are themselves a vulnerable population. Their emotional involvement and long-term commitment can lead to a decline in their own health and wellbeing (Sardas et al., 2018; Scarcella and Lonati, 2010). Prolonged caregiving responsibilities may lead to decreased wellbeing, as caregivers face physical fatigue, social isolation, and a lack of both family and service support (Les proches aidants ou des solidarités en action, 2019; Sasso, 2005; Schulz and Eden, 2016). Additionally, financial limitations and changes in family dynamics can create stress, and caregivers often struggle to adapt to shifting relationships with their loved ones as their role becomes more central (Meleis, 2010; Totman et al., 2015).

Caregivers also need comprehensive information about their loved one’s condition, especially regarding symptom management and medication administration (Funk et al., 2015; Wilson et al., 2018). Proper guidance from healthcare professionals is essential to fulfilling their caregiving role (Sheehy-Skeffington et al., 2014). However, the sense of isolation and anxiety that many caregivers feel is significant, particularly concerning end-of-life care and the lack of preparation for bereavement (Soroka et al., 2018; Mason and Hodgkin, 2019).

In conclusion, despite the unique nature of each caregiver’s role over time, broad areas of activity characterize family caregiving. This caregiving spans from assistance with daily activities and direct care provided to the recipient, to managing complex healthcare and social service systems (Schulz and Eden, 2016).

The pursuit of quality in the support provided to caregivers raises the same questions as that provided to the vulnerable individuals they assist. We have shown that supporting caregivers is necessary in elderly care arrangements (Haute Autorité de Santé, 2014a). This support raises the question of evaluating the involvement of caregivers. Benyo et al. (2023) show in their systematic review that there is no “standard” for assessing the healthcare needs of caregivers of patients and that the literature is limited regarding the medical burdens they face. Bunn et al. (2017) highlight that the role and involvement of caregivers lack formal recognition. Caregivers are often limited by confidentiality concerns related to their access to necessary information for care management. They also face personal and logistical challenges, including understanding healthcare systems and balancing family or professional obligations (Bunn et al., 2017). Social support and formal networks, although essential, are often insufficient. The two authors conclude that future research is needed to evaluate the physical health and comorbidities of caregivers as well as their engagement in the healthcare system to guide the subsequent implementation of support models to meet the needs of this population. In this context, the first question is whether the available assessment tools for family caregivers are relevant given the complexity of their role and whether they are scientifically validated (Pepin and Hébert, 2020).

Patients live longer than in the past, with chronic and often disabling illnesses. Recent treatments and the evolution of health policy have led to a shift from hospital care to home care (“Agir pour les aidants”, 2023). At home, a significant portion of the care for sick patients is provided by relatives rather than professional caregivers (Mace and Rabins, 2017).

• The role of caregiver requires availability, effort, and involves expenses necessary for access to professional help and even for the acquisition of specific equipment (Mace and Rabins, 2017)
• These developments thus impose significant demands, often referred to as “burdens,” on caregivers and the entire informal support network.

### Objectives

Our review aims to identify the various measurement tools related to family caregivers, determine the axes and dimensions explored, and assess their clinimetric properties.

## Methods

### Search strategy

A comprehensive literature search was conducted in the databases Google Scholar, ScienceDirect, Cairn, and PubMed using a combination of keywords from the following groups: (1) “familialities” OR “familiality” OR “familially” OR “familials” OR “familie” OR “family” [MeSH Terms] OR “family” OR “familial” OR “families” OR “family s” OR “familys”) AND “caregiver”; (2) “burden” OR “burdened” OR “burdening” OR “burdens”; (3) “aged” [MeSH Terms] OR “aged” OR “elderly” OR “elderlies” OR “elderly s” OR “elderlys”; (4) “stress” OR “stressed” OR “stresses” OR “stressful” OR “stressfulness” OR “stressing”; (5) “health services needs and demand” [MeSH Terms] OR (“health” AND “services” AND “needs” AND “demand”) OR “health services needs and demand” OR “needed” OR “needs” OR “needing”; (6) “assess” OR “assessed” OR “assessment” OR “assesses” OR “assessing” OR “assessment” OR “assessment s” OR “assessments”; (7) “quality of life” [MeSH Terms] OR (“quality” AND “life”) OR “quality of life” OR (“life” AND “quality”) OR “life quality”. To clarify our methodological approach, we targeted the MEDLINE, PsycINFO, and CINAHL databases, as they are recognized for their comprehensive coverage of literature in the fields of medicine, psychology, and nursing, respectively. Given that our review focuses on the burden and needs of family caregivers of older adults, these databases provide a broad collection of relevant studies. Additionally, our search strategy included Google Scholar and Cairn, the latter being particularly relevant for French-language literature, considering the context of our research team. This selection allowed us to maximize the likelihood of identifying a comprehensive range of assessment tools relevant to our topic. The search was limited to articles published in English or French up to January 2024. Titles and abstracts of search results were screened against the inclusion/exclusion criteria. The full text of relevant articles was reviewed, and those meeting the study criteria were included for further analysis. References were checked to identify additional articles that could potentially be included in the study.

### Study selection criteria

Articles were reviewed based on the following inclusion/exclusion criteria: (1) Family caregivers as the subject of the study. (2) Evaluation of their involvement, needs, or any other variable related to their tasks and activities. (3) The assisted persons are elderly individuals, with or without pathologies, limitations, or neurological disorders. (4) Data analysis using traditional or non-traditional grids or questionnaires.

Articles meeting the inclusion and exclusion criteria were reviewed for their contribution to the evaluation and analysis of family caregivers. Measurement methods were studied in detail, and the parameters considered were identified.

### Data extraction

After selecting the full-text articles based on the criteria, a primary reviewer (ELT) conducted the data extraction. Partial data extraction was also performed by an additional reviewer (ALG). The results were verified by (PP and AS). The clinical application and relevance were discussed and confirmed by the authors. Data extraction focused on three main areas: the characteristics of the dimensions assessed in family caregivers, the clinimetric qualities of the evaluation tools used in the studies, and the elderly populations involved with family caregivers. Characteristics included social, psychological, and organizational dimensions. The clinimetric elements of the tools used focused on: (1) Reliability through intraclass correlation coefficients or simple or weighted Kappa coefficients, internal consistency through Cronbach’s alpha or other coefficients. (2) Validity through different methods, including criterion validity, construct validity, and predictive validity.

## Results

### Search results

**FIGURE 1:**
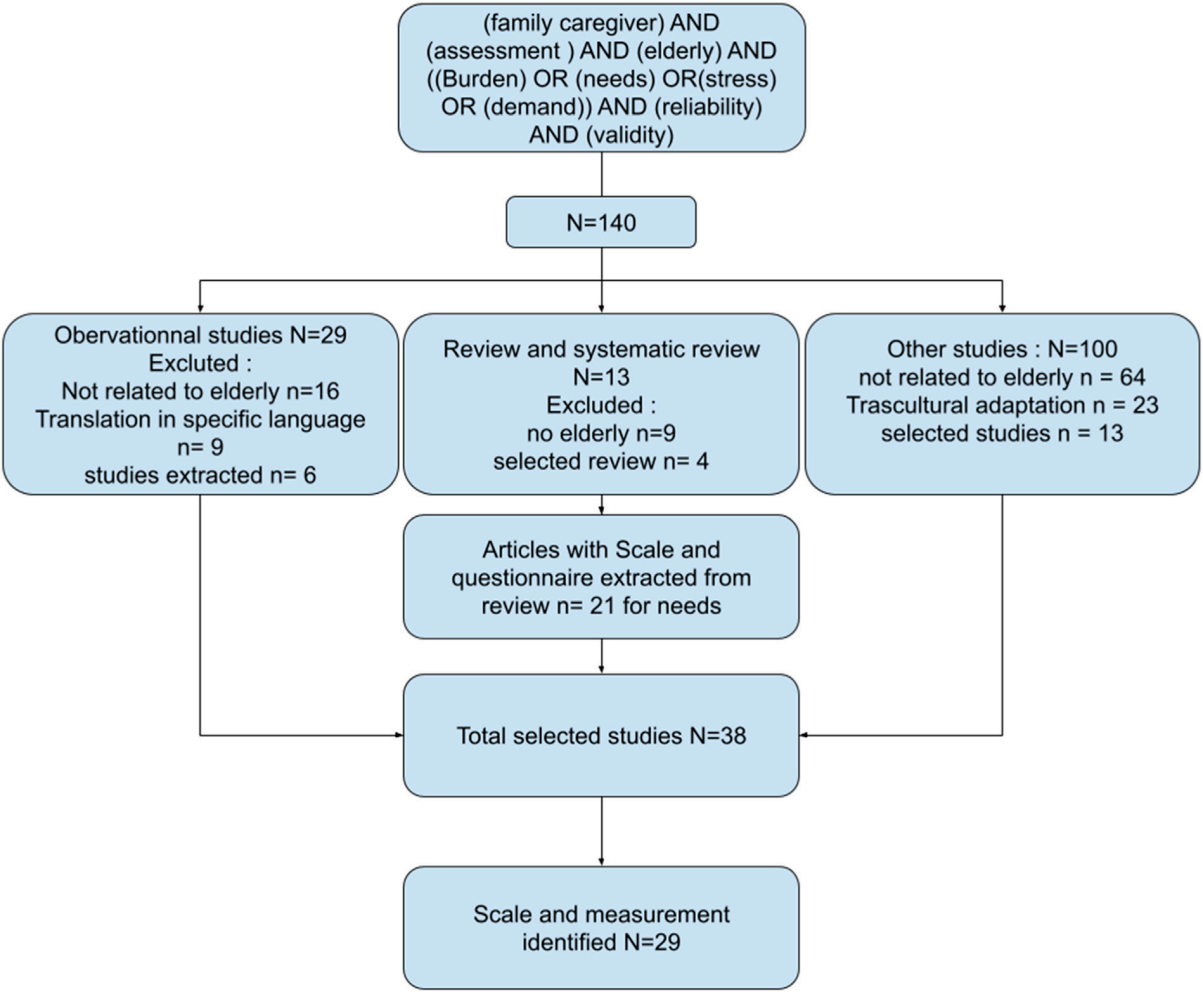
Flow chart, articles selection.

The search conducted on databases using the query “family caregiver” AND “assessment” AND “elderly” AND ” (burden OR needs OR stress OR demand)” AND “reliability” AND “validity” resulted in the identification of 140 articles. These included 13 reviews or systematic reviews, 29 observational studies, and 100 other types of studies. Articles that did not focus on elderly individuals or that involved cross-cultural adaptation studies were excluded. Following this exclusion process, 36 articles were retained. Specifically, 21 were from reviews or systematic reviews, 6 were from observational studies, and 19 fell into other categories. Collectively, these articles employed 29 scales, including two abbreviated scales, to assess different aspects of caregiver burden and support (Figure 1).

The retained studies evaluated various dimensions of caregiving through the use of these scales. A total of 17 scales assessed subjective burden (e.g., Zarit et al., 1980; Zhi-Xiang et al., 2023; Novak and Guest, 1989), while 9 scales focused on objective burden (e.g., Graßel et al., 2003; Schlomann et al., 2021). Stress was evaluated by 7 scales (e.g., Gerritsen and van der Ende, 1994; Peipert et al., 2018), and 4 scales assessed caregiver needs (e.g., Laprise et al., 2001; Cueli Arce et al., 2023). Additionally, 4 scales measured positive aspects of caregiving (e.g., Kate et al., 2012; Pendergrass et al., 2023). One scale assessed wellbeing. This is an instrument developed to assess the impact of informal caregiving on caregivers (Cejalvo et al., 2025). It comprises two components: the CarerQol-7D, which captures the subjective burden of caregiving, and the CarerQol-VAS, which reflects caregivers’ overall wellbeing.

Other aspects were measured by fewer scales: distress was evaluated by one scale (Kaufer et al., 1998; Cueli Arce et al., 2023), while 3 scales assessed caregivers’ control over the caregiving situation (e.g., Lim et al., 2022). Conflicting aspects were measured by 2 scales (O'Malley and Qualls, 2022), reactions to caregiving by 1 scale (Jaracz et al., 2022), and quality of life by another 1 scale (Matsuda, 1999). Further, scales were used to evaluate depressive symptoms (Peipert et al., 2018), expectations (Laprise et al., 2001), available resources (Li et al., 2023), and satisfaction with caregiving (Cueli Arce et al., 2023).

It was observed that most of the scales utilized in the studies measured the subjective burden of caregivers, though the understanding of this concept varied across scales. Objective burden was the second most commonly evaluated dimension. A summary of the scales and their characteristics is provided in Table 1.

**TABLE 1:** List of scales related to family caregivers.

Scale name	Dimensions	Nb items
Zarit Burden Interview (Zarit, Reever and Back-Peterson, 1980)	SB	22
Zarit Burden interview (short) (Zhi-Xiang, Lim and Chan, 2023)	SB	9
Caregiver Burden Inventory (Novak and Guest, 1989)	SB	24
Care giving burden scale (Gerritsen and van der Ende, 1994)	SB/S	20
Relative S scale (Greene et al., 1982)	S	15
Neuropsychiatric Inventory (Kaufer et al., 1998)	D	12
Burden Scale of Family Caregivers (Graßel, Chiu and Oliver, 2003)	OB	28
Burden Scale of Family Caregivers (short) (Graessel et al., 2014)	SB	10
Screen for caregiver Burden (Vitaliano et al., 1991)	SB	25
Berlin Inventory of Caregiver S-Dementia (Schlomann et al., 2021)	SB/OB/N/PA/CA	25
Subjective Burden of Caregivers of Demented Patients (SBS) (Matsuda, 1999)	SB/OB/S/QoL	14
Interview schedule (Pai and Kapur, 1981)	SB/OB	24
Self-Perceived Pressure (Pot, van Dyck and Deeg, 1995)	SB	9
Caregiver Burden Scale (Farley et al., 2008)	SB/OB	14
Carers Assessment of Difficulties Index (Charlesworth, Tzimoula and Newman, 2007)	SB/S	30
(Caregiver Questionnaire (Cole et al., 2014)	SB/OB	12
The Dementia Burden Scale (Peipert et al., 2018)	S/D	34
Echelle EAC (Laprise, Dufort and Lavoie, 2001)	N/E	28
Caregiver reaction assessment (CRA) (Given et al., 1992)	SB/OB	2
Multidimensionnal caregiver Strain index (MCSI) (Thornton and Travis, 2003)	S	13
Scale for Positive Aspects of Caregiving Experience (Kate et al., 2012)	PA	50
Caregiver Needs and Resources Assessment (Li et al., 2023)	D/R	36
Benefits of Being a Caregiver Scale (Pendergrass et al., 2023)	PA	14
Pearlin Mastery Scale among Family Caregivers (Lim et al., 2022)	C	7
Revised caregiving appraisal scale (RCAS) (Cueli Arce et al., 2023)	C/SA	25
The Caregiver Reaction Scale (O'Malley and Qualls, 2022)	SB/OB/N/D/PA/AC	40
Caregiver Burden Scale in Polish Caregivers of Stroke (Jaracz et al., 2022)	SB/OB/S/RC	22
CarerQol Instrument (Hoefman et al., 2013)	QoL	7

SB, Subjective burden; OB, Objective burden; S, Stress; D, Distress; N, Need; PA, Positive aspect; CA, Conflicting aspects; QoL, quality of life; E, expectation; R, ressources; C, Control; SA, Satisfaction; RC, Reaction.

### Clinimetric quality of the studies

The clinimetric quality of the studies was evaluated using an abbreviated version of the Consensus-based Standards for the Selection of Health Measurement Instruments (COSMIN, 2025) checklist. This tool assesses the methodological quality and performance of patient-reported outcome (PRO) instruments based on psychometric properties reported in validation studies. For the purposes of this study, the checklist was adapted to focus specifically on the criteria for reliability and validity.

Reliability, according to the COSMIN (2024), includes internal consistency, which was measured using Cronbach’s alpha. A study was considered to demonstrate high internal consistency reliability if Cronbach’s alpha was 0.80 or higher. The stability of responses over time was assessed through test-retest procedures. For continuous variables, intraclass correlation coefficients (ICCs) were used, while the Kappa coefficient was employed for categorical variables. It is important to note that simple correlation coefficients or two-factor analysis of variance were not considered compliant with current recommendations (COSMIN, 2024; Piette, 2016) and were annotated as non-compliant (NC). For reproducibility, we distinguished studies that did not conduct test-retest procedures by marking them as “NA” (not available) in the “retest” column of Table 2.

**TABLE 2:** List of scales related to family caregivers, psychometric quality.

Scale name	Consistency	Retest	Sample	Validity	Responsiveness
Zarit Burden Interview (Zarit, Reever and Back-Peterson, 1980)	Alpha	NA	>500 (Excellent)	y	NA
Zarit Burden interview (short) (Zhi-Xiang, Lim and Chan, 2023)	Alpha	NA	>200 (Excellent)	y	NA
Caregiver Burden Inventory (Novak and Guest, 1989)	Alpha	NA	<200 (Good)	nc	NA
Care giving burden scale (Gerritsen and van der Ende, 1994)	Alpha	NA	<200 (Good)	y	NA
Relative S scale (Greene et al., 1982)	Factor an	NA	<200 (Good)	NA	NA
Neuropsychiatric Inventory (Kaufer et al., 1998)	NA	NA	<200 (Good)	nc	NA
Burden Scale of Family Caregivers (Graßel, Chiu and Oliver, 2003)	Alpha	NA	<200 (Good)	y	NA
Burden Scale of Family Caregivers (short) (Graessel et al., 2014)	Alpha	NA	>200 (Excellent)	y	NA
Screen for caregiver Burden (Vitaliano et al., 1991)	Alpha	NA	<200 (Good)	nc	NA
Berlin Inventory of Caregiver S-Dementia (Schlomann et al., 2021)	Alpha	NA	>500 (Excellent)	y	NA
Subjective Burden of Caregivers of Demented Patients (SBS) (Matsuda, 1999)	Alpha	r	>200 (Excellent)	y	NA
Interview schedule (Pai and Kapur, 1981)	Intercor	NA	<50 (Poor)	nc	NA
Self-Perceived Pressure (Pot, van Dyck and Deeg, 1995)	Rho	NA	<20 (Fair)	nc	NA
Caregiver Burden Scale (Farley et al., 2008)	Alpha	ICC	<50 (Poor)	y	NA
Carers Assessment of Difficulties Index (Charlesworth et al., 2007)	Alpha	NA	>200 (Excellent)	nc	NA
Caregiver Questionnaire (Cole et al., 2014)	Alpha	ICC	≥200 (Excellent)	nc	NA
The Dementia Burden Scale (Peipert et al., 2018)	Omega	NA	>500 (Excellent)	y	NA
Echelle EAC (Laprise, Dufort and Lavoie, 2001)	Alpha	NA	>200 (Excellent)	y	NA
Caregiver reaction assessment (CRA) (Given et al., 1992)	Factor an	NA	>200 (Excellent)	y	NA
Multidimensionnal caregiver Strain index (MCSI) (Thornton and Travis, 2003)	Alpha	ICC	<200 (Good)	y	NA
Scale for Positive Aspects of Caregiving Experience (Kate et al., 2012)	Alpha	ICC	>200 (Excellent)	nc	NA
Caregiver Needs and Resources Assessment (Li et al., 2023)	Factor an	NA	>200 (Excellent)	nc	NA
Benefits of Being a Caregiver Scale (Pendergrass et al., 2023)	Alpha	NA	>500 (Excellent)	nc	NA
Pearlin Mastery Scale among Family Caregivers (Lim et al., 2022)	Omega	NA	>200 (Excellent)	nc	NA
Revised caregiving appraisal scale (RCAS) (Cueli Arce et al., 2023)	Alpha	NA	<200 (Good)	y	NA
The Caregiver Reaction Scale (O'Malley and Qualls, 2022)	Alpha	NA	>200 (Excellent)	nc	NA
Caregiver Burden Scale in Polish Caregivers of Stroke (Jaracz et al., 2022)	Alpha	Kappa	>200 (Excellent)	y	NA
CarerQol Instrument (Hoefman et al., 2013)	NA	NA	<500 (Good)	y	NA

y, yes; n, no; nc, non-compliant with recommendations; NA, not avalaible, Factor an., Factor analysis; Intercor, intercorrelation; Alpha, Cronbach alpha coefficient; ICC, intra classe correlation coefficient; Excellent, Good, Poor, Fair according to the COSMIN, checklist.

Validity, on the other hand, refers to the extent to which a PRO instrument accurately measures the construct it is intended to assess, as defined by the COSMIN (2024). Validity was typically confirmed by examining correlations with other relevant assessments.

Across the studies reviewed, a total of 19 reported Cronbach’s alpha values to evaluate internal consistency, with coefficients ranging from 0.53 to 0.99, depending on the specific questionnaire used. Two studies report omega values, three use factor analysis, two do not report any indices, and two use simple correlation.

Only four studies provided intraclass correlation coefficients (ICCs) to assess test-retest reliability, ensuring the stability of responses over time. Regarding the abbreviated versions of the Caregiver Burden Instrument (ZBI-22), Cejalvo et al. (2024) reported that most measures demonstrated satisfactory content and construct validity, with high internal consistency. However, they also noted that measurement invariance, criterion validity, and test–retest reliability were not consistently established across all versions. Additionally, structural validity was insufficient for certain versions. The authors conclude that both research and clinical practice would benefit from a standardized approach to improve the accuracy and consistency of caregiver strain assessment. Concern (Hoefman et al., 2013) among global population, concluded that the instrument demonstrated acceptable convergent, clinical, and discriminant validity; however, the average reliability coefficients were modest, with Cronbach’s alpha reported at 0.67 (95% CI [0.56, 0.75]) based on only 7 out of 54 studies. Test–retest reliability was reported in only three studies, with a pooled coefficient of 0.62 (95% CI [0.04, 0.89]). Among these three studies, Vluggen et al. (2021) reported an intraclass correlation coefficient (ICC) of 0.41 (95% CI [0.28, 0.53]). These values, all below the commonly accepted threshold of 0.7, suggest a moderate level of reliability and underscore a high degree of heterogeneity across studies.

About CRA (Caregiver reaction assessment) Luo et al. (2025) revealed acceptable internal consistency for the CRA scale, with Cronbach’s alpha values ranging from 0.76 to 0.79. Based on COSMIN standards, one version of the CRA scale is recommended for use, 14 versions are weakly recommended and six versions do not meet validity or consistency standards.

Additionally, 12 studies evaluated the validity of the instruments by reporting correlation coefficients with other measures, demonstrating the instruments’ capacity to measure the intended constructs.

This detailed analysis reveals variations in the reported levels of reliability and validity across the studies, which are summarized in Table 2.

### Pathologies of elderly individuals and caregivers

Among the 29 questionnaires reviewed, 13 are related to neurological conditions associated with aging. Of these, 13 are specifically designed for individuals with dementia, including studies such as those by Zarit et al. (1980), Zhi-Xiang et al. (2023), Novak and Guest (1989), and Greene et al. (1982), among others. Additionally, five questionnaires are tailored to patients with Alzheimer’s disease, as reported in studies by Novak and Guest, 1989, Kaufer et al. (1998), and Vitaliano et al. (1991). One questionnaire specifically addresses patients with Parkinson’s disease, as found in Greene et al. (1982). Moreover, 12 questionnaires target psychogeriatric patients, with examples from studies such as Gerritsen and van der Ende, 1994, Pai and Kapur, 1981, and Montgomery et al. (2000).

In addition to the above, two questionnaires are designed for patients who have suffered a stroke, as seen in studies by Greene et al. (1982) and Jaracz et al. (2022). Another questionnaire is focused on brain diseases, as reported by Novak and Guest, 1989. Three questionnaires address the needs of patients with physical disabilities, such as those in the studies by Lim et al. (2022), Cueli Arce et al. (2023), and O'Malley and Qualls, 2022. Lastly, 10 questionnaires are aimed at assessing patients who are losing autonomy or are dependent on care, with relevant studies including Montgomery et al. (2000), Peipert et al. (2018), and Laprise et al. (2001). This distribution reflects the range of neurological and physical conditions these questionnaires are designed to assess, offering a broad perspective on the different health states related to aging and caregiving.

## Discussion

The objective of this review was to identify tools to measure the consequences of family caregivers’ involvement, given their essential role in supporting and maintaining vulnerable individuals at home. Family caregivers play a crucial role, often at the expense of their own personal and professional lives, as well as their health and wellbeing. Their involvement is pivotal to the quality of life of the people they care for, especially those with dementia, as shown by several studies. Our review highlights issues with the clinimetric qualities of the tools intended for caregivers. Furthermore, some dimensions are not sufficiently assessed to develop appropriate support models and meet the specific needs of caregivers.

### Psychometric qualities of the scales

Our review highlights significant scientific weaknesses in the validation of the tools studied. In terms of internal consistency, while 26 studies report a Cronbach’s alpha greater than 0.8, some scales contain too many items, making them impractical for clinical use. This raises questions about the true value of internal consistency (Graßel et al., 2003; Cole et al., 2014; Peipert et al., 2018; Kate et al., 2012; Pendergrass et al., 2023; O'Malley and Qualls, 2022). Cortina (1993) demonstrated that the alpha coefficient is strongly influenced by the number of items. When a scale includes 40 or more items, it becomes relatively easy to achieve an acceptable alpha value (0.70 or higher) even if the average correlation between items is low or the scale is multidimensional. In such cases, a smaller subset of items may explain most of the variance, suggesting that shorter scales could maintain internal consistency and be easier to use in practice. However, two study uses the omega coefficient (Peipert et al., 2018), which is still debated regarding its relevance as a replacement for the alpha coefficient (Belan and Michelot, 2020).

A significant number of studies in our review failed to meet recommendations for test-retest reliability. To address this, intraclass correlation coefficients (ICCs) are recommended (Fitzpatrick et al., 1998). Furthermore, correlation coefficients only measure the strength of the association between two tests, not the level of agreement between them (Bland and Altman, 1986).

Regarding validity, 14 studies failed to comply with international methodological standards. Additionally, no studies reported data on the responsiveness of the scales (Kirshner, 1991). An instrument may be reliable and valid but still lack responsiveness, making it challenging to assess the change in the dimension being measured (Bland and Altman, 1986).

In summary, only two scales (Matsuda, 1999; Thornton and Travis, 2003) demonstrate sufficient reliability, strong correlations, and validated conceptual and predictive validity for family caregivers. However, both have limitations. The first was tested only in a population with dementia, limiting its generalizability. The second focuses solely on stress and does not provide enough relevant information on other dimensions. Further research is needed to validate these instruments in more diverse contexts.

### The impact of caregiving on health

The main health issues linked to caregiving include stress (38%), disturbed sleep (32%), and physical pain (30%). According to the 2018 BVA-Fondation April survey, 31% of caregivers neglect their own health due to their caregiving responsibilities. Studies show that caregivers experience significantly higher levels of stress and depression, along with lower levels of subjective wellbeing, physical health, and self-efficacy compared to non-caregivers (Les proches aidants ou des solidarités en action, 2019). These differences are particularly significant for stress, self-efficacy, depression, and subjective wellbeing, though less pronounced for physical health. This may be due to the immediate negative effects of caregiving stress and the difficulty of maintaining a sense of competence when faced with complex care management and little control over the care recipient’s symptoms.

As a result, 40% of caregivers with a high burden feel depressed, which is eight times higher than those who do not feel burdened. More than half of those heavily burdened also suffer from sleep disorders and back problems. These caregivers use psychotropic drugs more frequently than other caregivers (Sherwood et al., 2005). The impact of caregiving on depression may stem from chronic stress fatigue (Teel et al., 1999), as well as psychological responses to long-term caregiving demands. Clinical measures of depression, which are less prone to concealment than self-reports, further highlight these effects (Vitaliano et al., 2005).

Caregivers also report nearly double the rates of heart disease, cancer, diabetes, and arthritis compared to non-caregivers. Some research shows that caregivers experience a weakened immune response, leading to more infections and higher cancer risks. Physical tasks like helping someone get up, bathe, or walk increase the risk of discomfort, strain, pain, and backache (Buyck et al., 2013). A global point-biserial analysis using BESD shows a correlation of 0.09, indicating a 9% higher risk of health problems for caregivers compared to non-caregivers with the same demographic profile. Caregivers of patients with conditions such as Alzheimer’s, vascular dementias, and Parkinson’s face similar challenges due to the specific demands of these diseases (Vitaliano et al., 1991). Caregiving thus represents a chronic stress, defined by the personal identification as a caregiver and the cognitive, functional, and emotional deficits of the care recipient (Vitaliano et al., 1991).

In addition, a study highlights the lack of attention caregivers receive from medical professionals. Only 13% of caregivers report being asked about their health when accompanying their loved ones to the hospital, suggesting there is still much to be done to raise awareness on this issue (Les proches aidants ou des solidarités en action, 2019).

### The importance of prevention for family caregivers: reducing stress and improving wellbeing

The need for prevention for family caregivers also highlights the significant effects they face and the importance of early intervention to mitigate these effects. As mentioned in the previous chapter, family caregivers face substantial levels of stress, depression, and a noticeable deterioration in overall wellbeing, underscoring the vulnerability of this demographic group.

It is recommended to use community strategies such as support groups and respite services, which play a crucial role in providing emotional and practical support to family caregivers. Research by Paggi (Haute Autorité de Santé, 2014b) emphasizes that these initiatives help reduce psychological distress and the risk of burnout among caregivers while fostering a supportive environment. Additionally, prevention programs for family caregivers have been recognized for their economic benefits, reducing healthcare costs and optimizing the use of available social and community resources (Brodaty and Donkin, 2009).

Investing in prevention for family caregivers is therefore essential not only for improving their quality of life and wellbeing but also for building a more resilient and sustainable socio-economic society. These interventions do not merely mitigate the negative effects of caregiving on the individuals involved but also address the growing needs of caregivers, whose role is crucial in the context of an aging population and increasing pressures on healthcare systems. The perceived burden of caregivers is likely a determinant of their health that needs to be better analyzed to move towards identification and personalized support that closely meets their needs (Delbrouck et al., 2021).

### Burnout among family caregivers: an underestimated phenomenon

Burnout syndrome, defined as a response to chronic stress related to obligations or work, can develop into a long-term condition leading to various health problems (Delbrouk et al., 2021). Burnout is characterized by the gradual depletion of physical, emotional, and cognitive resources due to prolonged demands (Machado, 2015). Maslach and Jackson describe burnout as a psychological syndrome that includes emotional exhaustion, depersonalization, and a reduced sense of professional efficacy, often seen in healthcare professionals under intense pressure (Picart and Jaussaud, 2018).

Emotional exhaustion is marked by deep emotional fatigue caused by the psychological strain of continuous care (Montero-Marin and Garcia-Campayo, 2010). Burnout evolves in three stages: an initial overload, followed by repetitive tasks that seem fruitless, and finally, accumulated fatigue leading to avoidance behaviors (Montero-Marin and Garcia-Campayo, 2010). Although well-studied in healthcare workers, burnout is less explored among family caregivers, despite similar workloads and stress (Dyrbye et al., 2017). Both family and professional caregivers play essential roles in patient care, but their contexts differ. Family caregivers, often without formal training, provide care based on emotional bonds, adjusting their personal lives to care needs, often on a full-time basis (Dyrbye et al., 2017).

While both groups share the responsibility for quality care, family caregivers are rarely paid for their work, unlike professional caregivers. This difference also affects the support they receive. Professional caregivers benefit from institutional resources, while family caregivers may feel under-recognized and unsupported financially. However, McDarby et al. (2023) shows, there are already gaps among healthcare professionals who face dementia and end-of-life care in their role as professional caregivers. In family caregivers, emotional exhaustion leads to feelings of depletion and loss of control over outcomes, sometimes resulting in withdrawal from caregiving tasks (Maslach et al., 1997). As populations age and life expectancy increases, caregivers themselves are often elderly. For instance, Orfila et al. (2018), in a study involving 829 caregivers, reported a mean caregiver age of 63.3 years, with 80% being women. Care recipients in this cohort had an average age of 84.2 years. The study further revealed a heightened risk of mistreatment and health complications linked to caregiving responsibilities, with odds ratios ranging from 2 to 7 depending on the severity of the recipient’s impairment, as compared to non-caregivers.

It is crucial to measure burnout symptoms among caregivers to identify those in distress and provide immediate support. Targeted interventions can preserve their wellbeing, benefiting not only the caregivers but also the people they care for.

### Towards a nuanced evaluation of family caregivers’ burden: limits and perspectives of current measurement instruments

Most of the questionnaires analyzed focus on measuring subjective burden, with 16 scales addressing this concept. However, definitions and interpretations of burden vary widely, leading to inconsistencies across tools. Additionally, many of these questionnaires target specific pathologies such as dementia, Parkinson’s disease, and Alzheimer’s disease. The burden scales do not cover the same elements or items, which introduces heterogeneity and makes comparisons between scales difficult. For instance, despite the popularity of the Zarit scale, no studies have examined its specific impact on the doctor-patient relationship (Vitaliano et al., 1991). Moreover, there is a lack of research guiding practitioners on how to act based on the scores obtained from such questionnaires (Gibert, 2015). No standardized recommendations exist to help clinicians choose the most appropriate scale for a given situation (Gibert, 2015). It is even suggested that administering the Zarit scale could induce stress in caregivers, as they are confronted with their own challenges during the process (Vitaliano et al., 1991). This paradox raises the question of whether measuring burden truly benefits caregivers or, in some cases, exacerbates their stress.

There is a widespread assumption that caregiving negatively impacts caregivers’ physical and mental health, increasing their morbidity and mortality (Amieva et al., 2012). However, research shows a more nuanced reality. While about one-third of primary caregivers report negative effects on their physical or mental health, an equal proportion indicates positive effects from their caregiving role (Soullier, 2012). Recent literature supports this, with an English study revealing that 95% of caregivers derive satisfaction from providing care (Bamford et al., 1998).

The terminology used in this field also requires urgent reassessment. The term “burden” may be overly reductive when evaluating the caregiver experience. Some authors advocate for the use of “caregiver load” instead of “burden” to avoid confusion and to better reflect the multifaceted nature of caregiving (Bamford et al., 1998). As caregiving practices evolve and the role of family members as care partners becomes more central, it is essential to develop tools that better align with caregivers’ needs. Currently, very few questionnaires (8 in total) have been validated and translated into French, complicating the efforts of practitioners to assess and support caregivers effectively. This lack of tools also perpetuates the presumption of burden, which can have serious implications for caregivers’ health.

In summary, assessing caregiver burden should not be limited to the use of standardized instruments. A more nuanced approach, tailored to specific caregiving contexts, is essential to meet the complex and evolving needs of family caregivers. In conclusion, the reconsideration of the term “burden” is motivated by several observations emerging from our review and the broader literature. First, definitions and interpretations of burden vary significantly across assessment tools, introducing heterogeneity and limiting comparability. Second, the term “burden” may be overly reductive and fail to capture the multidimensional nature of the caregiving experience. Some authors advocate for alternative terms such as “caregiver load” or “weight of caregiving” to better reflect this complexity. Such a shift in terminology could influence how results are interpreted by placing greater emphasis on the overall impact of caregiving, including both its negative and potentially positive dimensions.

### Clinical and research perspective

From a clinical standpoint, assessing family caregivers is essential for preventing burnout and promoting caregiver well-being—two outcomes that directly influence the quality of care delivered to older adults and the long-term viability of informal caregiving systems. These findings underline the urgent need for assessment tools that encompass all relevant dimensions of the caregiving experience. The importance of family caregivers extends beyond individual households, bearing significant weight in the healthcare system. According to Petty (2015), family caregivers provide services equivalent to $450 billion annually in the U.S. economy. Recent studies have emphasized the necessity of structured support programs tailored to caregivers’ needs. Although these programs vary widely in scope and format, the growing body of evidence supports their continued development (Bongelli et al., 2024; Alam et al., 2020; Yang et al., 2024; Petty, 2015; Abdul Wahab et al., 2024). However, a critical analysis of current assessment tools reveals psychometric limitations that must be considered carefully in clinical and research applications. Based on our review, several recommendations emerge.

First, there is a need to develop a caregiver-specific assessment instrument, drawing inspiration from existing validated questionnaires. It is essential to measure and identify symptoms of burnout in order to detect those in acute distress who require immediate support. In this context, adapting the Maslach Burnout Inventory (Kinman, 2025)—originally designed for healthcare professionals—may be appropriate to capture the emotional and contextual nuances specific to family caregivers, who often face emotional over-involvement and institutional isolation.

Additional conceptual grounding could be drawn from instruments such as the Adult Social Care Outcomes Toolkit (ASCOT; Rand et al., 2015), which evaluates quality-of-life outcomes related to long-term social care services. Second, the development of such a tool should follow rigorous methodological guidelines, specifically the COSMIN (COnsensus-based Standards for the selection of health Measurement INstruments) framework. This would ensure robust evaluation of the tool’s psychometric properties, including content validity, reliability, responsiveness, and criterion validity, thereby supporting its scientific and clinical utility. Third, there is a pressing need to systematically expand support structures for family caregivers (Malki et al., 2025; Marinho et al., 2022; Bohrnstedt, 1983). These supports should be grounded in empirical evidence and tailored to the complex challenges this population faces—particularly in relation to emotional burden, social isolation, and physical and psychological health risks.

## Conclusion

Family caregivers play a vital, multidimensional role in the care of loved ones. Their involvement often requires significant adjustments to their daily lives, with many providing care without financial compensation. This can take a toll on their physical and mental health. Nonetheless, they are essential in ensuring the comfort and dignity of the elderly or sick.

A preventive approach to supporting family caregivers is necessary to maintain their physical, emotional, and social wellbeing. By anticipating the challenges they face, programs can be implemented to reduce the risks associated with caregiving and prevent burnout. Such efforts would promote sustainable caregiving relationships and enhance the quality of life for both caregivers and those they assist.

Our review also highlights the limitations of current scales used to measure caregivers’ engagement and resources. The concept of “burden” needs reevaluation, with a shift toward identifying and addressing caregivers’ specific “needs.” Developing a more nuanced and preventive approach would go beyond measuring burden and allow for personalized support.
